# Does financial compensation matter for research participation in prevention trials in Tanzania? Perspectives of healthy volunteers

**DOI:** 10.1371/journal.pone.0321353

**Published:** 2025-09-29

**Authors:** Raymond Athanas, Heavenlight A. Paulo, Gasto Frumence, Masunga K. Iseselo, Connie M. Ulrich

**Affiliations:** 1 Department of Bioethics and Health Professionalism, Muhimbili University of Health and Allied Sciences, Dar Es Salaam, Tanzania; 2 Department of Epidemiology and Biostatistics, Muhimbili University of Health and Allied Sciences, Dar Es Salaam, Tanzania; 3 Department of Biostatistics, School of Public Health, Boston University, Boston, Massachusetts, United States of America; 4 Department of Development Studies, Muhimbili University of Health and Allied Sciences, Dar Es Salaam, Tanzania; 5 Department of Clinical Nursing, Muhimbili University of Health and Allied Sciences, Dar Es Salaam, Tanzania; 6 Biobehavioral Department, School of Nursing, NewCourtland Center for Transitions and Health, University of Pennsylvania, Philadelphia, Pennsylvania, United States of America; 7 Leonard Davis Institute of Health Economics, University of Pennsylvania, Philadelphia, Pennsylvania, United States of America; 8 Department of Medical Ethics and Health Policy, Perelman School of Medicine, University of Pennsylvania, Philadelphia, Pennsylvania, United States of America; Delta State University, NIGERIA

## Abstract

**Background:**

Financial compensation is an important aspect of respect for people enrolled in clinical research, yet continues to raise ethical questions. Respondents’ characteristics, shaped by contextual and geographic factors, may influence perceptions of the ethical implications of financial compensation. This study explored the relationship between respondents’ characteristics and their ethical perspective on the role of financial compensation in prevention trials in Tanzania.

**Materials and methods:**

A cross-sectional survey of 537 participants from two prevention studies within Tanzania was conducted. Participants were eligible if they participated in either the HIV Pre-Exposure Prophylaxis Vaccine Trial (PrEPVacc) trial or a non-inferiority trial of low-dose compared to standard high-dose calcium supplementation in pregnancy in Tanzania. A questionnaire was used to collect participants’ sociodemographic characteristics and motivating reasons for research participation. Data analysis was performed using descriptive statistics for all variables. The chi-square test was used to assess associations between categorical demographic characteristics and binary outcomes scores. Modified Poisson regression models were employed to examine predictors of positive or negative perspectives for each outcome. The P value of < 0.05 was used to ascertain the significant association between the variables.

**Results:**

Most participants (82.5%) were young adults between the ages of 18–28 years, married or cohabiting (60.7%), and self-employed (55.9%). All participants in the cohort were female. Key motivations for research participation included medical follow-up (96.8%), quality information (94.4%), and disease control (92.2%). The majority also reported feeling a duty to participate (90.5%) with 50.1% indicating that they felt an obligation to the person who requested their participation. More than a third (36.9%) wanted to receive an incentive and reimbursement (38.7%) for out-of-pocket expenses. Financial compensation was a less common motivator among married or cohabiting participants compared to single individuals (APR = 0.54, 95% CI: 0.42–0.71, p < 0.001). Participants with primary education were also less likely to report financial compensation motivations compared to those with no formal education (APR = 0.57, 95% CI: 0.41–0.78, p < 0.001). Also, participants who had taken part in two or more trials had significantly higher non-financial participation motivations compared to those with only one research experience (p < 0.001).

**Conclusion:**

Financial compensation matters to certain participant subgroups, but its role varies. While some value monetary benefits, others prioritize altruism and trust in research oversight. A balanced, transparent compensation framework is needed to address financial needs while maintaining ethical integrity.

## Introduction

Financial compensation for research participants is a complex ethical issue in clinical trials, particularly in low- and middle-income countries (LMICs). While financial compensation is intended to acknowledge participants’ time and effort [1,2], concerns about undue inducement and exploitation remain [3,4]. The ethical implications of financial compensation are influenced by socio-demographic factors, yet the extent to which these factors shape participants’ ethical perspectives is not fully understood. Age is a significant determinant of ethical viewpoints on financial compensation. Younger participants, particularly those facing economic instability, may be more financially motivated, while older individuals often prioritize non-financial benefits, such as access to healthcare or altruistic contributions [3,5–7]. However, the role of age in shaping ethical reasoning around financial compensation remains under-explored [8].

In Tanzania, familial ties play a crucial role. Financial compensation in research studies may be perceived through this lens, influencing household support and family dynamics. Marital status and household dynamics may shape ethical perspectives on research participation and the role of financial compensation, with married participants potentially viewing compensation as a necessity and a benefit [9]. In their study, Lobato et al. reported gender differences in research participation where women were not only more likely to participate in research based on altruistic considerations but also rely on their male counterparts [10]. Women are generally more inclined than men to perceive participants as being unduly induced when a payment offer influences their decision to participate, despite their initial reluctance [11].

Some studies have shown that educational level plays a role in informed consent depending on the type of disease [12]. Lower educational levels of participants can increase the risk of misunderstanding trial protocols, raising ethical concerns about informed consent and the potential for exploitation, especially where financial incentives are involved [4]. Meanwhile, occupational status, income level, and financial dependence influence how compensation is perceived, yet research has not adequately examined how financial insecurity in LMICs affect trial participation decisions [13]. Low-income participants may view financial compensation as a necessity rather than a supplemental to the trial, increasing concerns about undue inducement, whereas higher-income participants may prioritize compensation related to risk and long-term security [3,6,8,9]. Moreover, prior research experience can shape participants’ expectations of financial compensation, but little is known about how experience modifies ethical considerations, particularly regarding injury compensation and long-term benefits [8].

Thus, in the context of Tanzania and the many different geographical locations within the country, we do not know if respondents’ socio-demographic characteristics affect their views on research participation in prevention trials and whether financial and other non-financial motivations such as altruism, receiving up-to-date information, or controlling one's disease are more important for their participation. This study examined the association between healthy volunteers’ socio-demographic characteristics and their ethical perspectives on the role of financial and non-financial compensation motivations in prevention trials in Tanzania.

## Materials and methods

### Study design, setting and population

This cross-sectional study was conducted as an adjunct to two ongoing prevention trials conducted in Tanzania from 20^th^ June 2022–21^st^ April 2023 among healthy volunteers including the HIV Pre-Exposure Prophylaxis Vaccine Trial (PrEPVacc) trial (completed in July 2024), and the non-inferiority-of-low-dose-compared to standard high-dose calcium supplementation in pregnancy: a randomized, parallel-group, non-inferiority trials in Tanzania (completed in December 2023) [14]. The survey was part of a larger multi-method study on financial compensation in prevention trials in Tanzania [15]. Participants were eligible if they participated in one of the two prevention trials that were ongoing in Tanzania (PrEPVacc or calcium supplementation). Participants in the PrEPVacc Trial were eligible if they met the following criteria: aged 18 to 45 years, identified as female sex workers (FSWs), and tested HIV-negative at the time of enrollment. For the calcium supplementation group, eligible participants were pregnant women between the ages of 18 and 45. Both groups must have the capacity to provide informed consent, and individuals were excluded if they did not meet the inclusion criteria as stated.

### Sample size and sampling procedures

A total of 641 (100%) participants were initially recruited for the study: 274 PrEPVacc healthy volunteers from MUHAS, 217 from PrEPVacc at the National Institutes of Medical Research (NIMR)-Mbeya, and 150 calcium supplementation healthy volunteers from MUHAS. Of these, 104 declined participation, resulting in a final sample of 537 (83.8%) participants who provided informed consent and were successfully interviewed. The final distribution was as follows: 103 calcium supplementation volunteers (MUHAS), 241 PrEPVacc volunteers (MUHAS), and 193 PrEPVacc volunteers (NIMR-Mbeya). This sample size provided 80% power to detect associations between ethical perspectives on financial compensation and participants’ background characteristics, assuming a small-to-medium effect size at a 5% significance level.

### Survey development and measures

A self-report questionnaire was developed by the primary author (RM) with items from qualitative interviews and the existing literature, modified to align with the Tanzanian context [11,12]. The items were pretested with 10 healthy volunteers in the PrEP Vacc trial at MUHAS who were not part of the current study. Minor modifications were made for clarity including the monthly income variable. Items included motivations such as altruism, financial and non-financial, and socio**-**psychological.

#### Independent variables.

The socio-demographic characteristics of the participants included age, marital status, education level, occupation, number of dependents, participation experience, and monthly income. The gender of the participants is excluded from the list of socio-demographic characteristics since all were females.

#### Outcome measure.

The dependent variable reflects perspectives on research participation in prevention trials, including the role of financial compensation and other motivations. Items were measured on a 5-point Likert scale ranging from 1 (strongly disagree) to 5 (strongly agree) with higher scores yielding more favorable perceptions. Three distinct outcomes were derived from the 23 items: (1) financial compensation, comprising three financial-related items (α = 0.85); (2) non-financial motivations, comprising the remaining 20 items (α = 0.68); and (3) a full ethical perspective scale including all 23 items-financial and non-financial (α = 0.77). These variables reflect how respondents perceive, evaluate, or respond to the role of financial compensation and other non-financial motivations in their participation.

### Data collection and ethical considerations

Participants were recruited for the study with permission of the prevention trial principal investigators (PIs) and permission letters were requested and obtained from the PIs of the two prevention trials, as well as local and regional government authorities to access study participants. Each participant was provided with the written informed consent form (ICF) prior to their participation in the study, explaining the study's purpose, procedures, risks, benefits, confidentiality, voluntariness and their right to withdraw. All of whom were literate and able to review the consent form independently. Participants signed the written informed consent form, which was securely stored in accordance with institutional guidelines. Participants were informed that their participation in the study was entirely voluntary. They had the right to withdraw at any time without providing a reason and without any consequences. Additionally, participants were free to skip or decline to answer any specific question if they felt uncomfortable or preferred not to respond. The research assistant/s was present during the consent process to answer any questions and ensure understanding before participants signed the form. At some points, also one of the co-authors (GF) was present. Participants were interviewed in a private room and their information was strictly confidential. All data we securely stored in password-protected digital files and locked physical storage, accessible only to authorized researchers.

The first author (RM) assisted by two trained research assistants collected the data using interviewer-administered questionnaires to allow for completeness, clarifications and deeper insights. Participants were interviewed in a private setting to ensure confidentiality and minimize external influence. Efforts were made to create a neutral environment, ensuring participants felt comfortable providing honest responses and reducing response bias. Ethical clearance for this study was obtained from MUHAS Institutional Review Board (MUHAS-REC-02-2022-981) and the National Institute for Medical Research (NIMR) (NIMR/HQ/R.8a/Vol.IX/4547). The authors confirm that the study was performed in accordance with the principles in the Declaration of Helsinki. Data were collected between August 2022 to April 2023.

### Data analysis

All analyses were performed using Stata statistical software [16]. Statistical significance was assessed at a 5% level, with results presented in the form of summary statistics, association tests, and regression model outputs. We conducted a comprehensive statistical analysis to explore the characteristics of participants, examine relationships among variables, assess the reliability of measurement scales, and evaluate the psychometric properties of the data. Demographic characteristics were summarized using frequencies and percentages. Age was categorized into three groups to see if there would be differences by age group (18–28, 29–31, and 32 years and above), while other demographic factors were categorized and labeled for interpretability. The reliability of a 23-item scale assessing participant perspectives was evaluated using Cronbach’s alpha. Items with low reliability (<0.7) were iteratively removed to identify the most robust subset of items.

Furthermore, a graded response model (GRM) based on item response theory (IRT) was applied to examine item-level performance. Goodness-of-fit tests and item characteristic curves (ICCs) were assessed to ensure validity and consistency. For the three distinct outcomes, summative scores (total scores) for each outcome were calculated and dichotomized into positive and negative perspectives based on predefined thresholds.

The chi-square test was used to assess associations between categorical demographic characteristics and binary outcomes of the perspective scores. Modified Poisson regression models with robust standard errors were employed to examine predictors of positive or negative perspectives for each outcome (financial compensation perspective, non-financial compensation perspective, and the full-scale). Covariates included age, marital status, education, income, and occupation, adjusting for potential confounding effects.

## Results

The majority (82.5%) of participants were aged 18–28, with a mean age of 24.5 years (±4.4). A significant portion (60.7%) were married or cohabiting, while 37.4% were single. Nearly half of the participants had primary education (46.9%), followed by secondary education (45.1%). Participants with education beyond secondary level were minimal (4.3%). Over half (55.9%) were self-employed, with 34.4% being unemployed (Table 1).

**Table 1:** Sociodemographic characteristics of participants.

Characteristics	Number (n)	Percentage (%)
**Age group (Years)**		
18-28	443	82.5
29-31	50	9.3
32 and above	44	8.2
**Mean age (±SD)**	24.5 (4.4)	
**Marital status**		
Single	201	37.4
Married/cohabit	326	60.7
Divorced	10	1.9
**Education Level**		
No formal	20	3.7
Primary	252	46.9
Secondary	242	45.1
Above Secondary	23	4.3
**Occupation**		
Employed	52	9.7
Unemployed	185	34.4
Self-employed	300	55.9
**Number of dependents**		
None	37	6.9
1-2	298	55.5
3-4	144	26.8
5 and above	58	10.8
**Participation experience (**number of trials)		
1	473	88.1
2 and above	64	11.9
**Monthly income**		
0-$**59.82**	415	77.3
Above $**59.82**	122	22.7
**Ethical perspective**		
Negative	83	15.5
Positive	454	84.5
**Mean perspective score (±SD)**	80.3 (11.4)	

### Motivations for research participation in a prevention trial

Table 2 provides statistics and inferential tests for the 23 items assessing various motivations and perceptions regarding participation in a prevention trial. Participants reported high levels of agreement items (≥80%) with a mean score ≥4.0 and significant association (*IRT p-value* <0.001). Most participants indicated that they participated in prevention trials to receive medical follow-up (96.8%), the quality of the information they received from the principal investigators (94.4%), to help control their disease (92.2%), and a felt obligation to participate (90.5%). Interestingly, more than a quarter (29.1%) were influenced by family and friends to participate. A smaller but significant percentage of participants wanted to receive an incentive (36.9%), reimbursement for their time (38.7%) and compensation for any injury they might incur (29.6%).

**Table 2:** Motivations for participation in a prevention trial: Mean scores and agreement levels.

Item	Concepts and Items	Mean (SD)	Agree/ strongly agree (%)	IRT p-value#
A1	I felt the duty to participate in the prevention trial	4.27 (0.91)	90.5%	<0.001
A2	I felt an obligation to the person who requested my participation	2.88 (1.57)	50.1%	<0.001
A3	I wanted to be with a friend or family member who is participating	2.50 (1.62)	38.0%	<0.001
A4	I wanted to contribute to the advancement of science	4.02 (1.00)	80.6%	<0.004
A5	I wanted to contribute to the health of others	3.82 (1.19)	76.1%	0.529
A6	I was curious about the prevention trial project	3.69 (1.37)	73.4%	<0.001
A7	I wanted to receive an incentive (e.g., money)	2.57 (1.60)	36.9%	<0.001
A8	I wanted to participate in something important	4.14 (0.94)	85.1%	<0.001
A9	I wanted to have a new experience in the prevention trial	3.30 (1.46)	59.0%	<0.001
A10	I was influenced by my friends/family	2.23 (1.54)	29.1%	<0.001
A11	I knew that I would receive compensation for any injury resulting from the trial	2.37 (1.48)	29.6%	<0.001
A12	I have a personal connection to the project	3.86 (1.15)	75.6%	<0.001
A13	I felt that others will view my participation as something good	3.69 (1.13)	68.9%	<0.001
A14	I wanted to participate in the development of a new vaccine	3.92 (1.06)	78.8%	<0.001
A15	I wanted to help my community/society	4.13 (0.91)	87.7%	<0.001
A16	I wanted to help to control this disease/infection	4.31 (0.82)	92.2%	<0.001
A17	I wanted to receive reimbursement for my out-of-pocket expenses	2.68 (1.61)	38.7%	<0.001
A18	I wanted psychological benefits (e.g., feeling good about myself)	4.01 (0.95)	82.3%	<0.001
A19	I wanted to help neighbors/to protect others	4.05 (1.02)	84.9%	0.158
A20	I feel at risk for the disease	3.00 (1.68)	51.0%	<0.001
A21	The quality of information provided by the Investigators	4.48 (0.86)	94.4%	<0.001
A22	Medical follow-up planned for this prevention trial	4.61 (0.67)	96.8%	<0.001
A23	My previous experience on similar project	1.75 (1.29)	15.1%	<0.001

### Association between financial compensation and participants’ background characteristics

Financial compensation was a less common motivator among married or cohabiting participants compared to single individuals (APR = 0.54, 95% CI: 0.42–0.71, p < 0.001). Participants with primary education were also less likely to report financial compensation motivations compared to those with no formal education (APR = 0.57, 95% CI: 0.41–0.78, p < 0.001). Similarly, participants with secondary education were significantly less likely to report this outcome (APR = 0.61, 95% CI: 0.44–0.83, p = 0.002). The strongest negative association was found for participants with above secondary education (APR = 0.10, 95% CI: 0.01–0.76, p = 0.026). Moreover, participants who had taken part in two or more trials had a significant positive association in the crude analysis (PR = 1.64, 95% CI: 1.24–2.17, p < 0.001), indicating that more experienced participants were more likely to report agreement with financial compensation compared to those with less experience. However, the adjusted result (APR = 1.29, 95% CI: 0.96–1.73, p = 0.090) was not statistically significant (Table 3).

**Table 3:** Association between financial compensation motivations and participant background characteristics.

Characteristics	PR (95%CI)	P-value	APR (95%CI)	P-value
**Age group(years)**				
18-28	Ref		Ref	
29-31	1.22 (.85, 1.76)	0.282	0.78 (0.52, 1.18)	0.237
32 and above	0.76 (.45, 1.29)	0.318	0.61 (0.37, 1.02)	0.062
**Marital status**				
Single	Ref		Ref	
Married/cohabit	0.50 (0.40, 0.64)	<0.001	0.54 (0.42, 0.71)	<0.001
Divorced	0.85 (0.40, 1.8)	0.673	0.81 (0.41, 1.61)	0.555
**Education level**				
No formal	Ref		Ref	
Primary	0.56 (0.38, 0.84)	0.005	0.57 (0.41, 0.78)	<0.001
Secondary	0.54 (0.36, 0.80)	0.002	0.61 (0.44, 0.83)	0.002
Above Secondary	0.07 (0.01, 0.51)	0.008	0.10 (0.01, 0.76)	0.026
**Occupation**				
Employed	Ref		Ref	
Unemployed	0.76 (0.45, 1.30)	0.317	0.78 (0.45, 1.33)	0.364
Self-employed	1.54 (0.96, 2.45)	0.073	1.2 (0.75, 1.93)	0.445
**Number of dependents**				
None	Ref		Ref	
1-2	0.86 (0.52, 1.42)	0.551	0.97 (0.62, 1.51)	0.889
3-4	1.31 (0.79, 2.16)	0.298	1.54 (0.97, 2.45)	0.068
5 and above	1.06 (0.60, 1.91)	0.837	1.11 (0.62, 1.96)	0.729
**Participation experience (number of trials)**				
1	Ref		Ref	
2 and above	1.64 (1.24, 2.17)	<0.001	1.29 (0.96, 1.73)	0.090
**Income**				
0-$**59.82**	Ref			
Above $**59.82**	1.10 (0.83, 1.45)	0.503	1.08 (0.83, 1.4)	0.583

### Association between non-financial reasons and participant background characteristics

Divorced participants had significant positive non-financial compensation motivations association in the crude analysis (PR = 1.07, 95% CI: 1.03–1.11, p < 0.001) compared to single, married or cohabit participants. However, the adjusted result (APR = 1.03, 95% CI: 0.97–1.10, p = 0.337) was not statistically significant. Participants with education levels above secondary school had a significantly higher likelihood of a positive outcome for motivations beyond financial compensation compared to those with no formal education (APR (95% CI): 1.28 (1.01, 1.62). P-value: 0.045). Those with 2 or more research experiences also had significantly higher non-financial participation motivations compared to those with only one research experience (p < 0.001) (Table 4).

**Table 4:** Association between non-financial reasons and participant background characteristics.

Characteristics	PR (95%CI)	P-value	APR (95%CI)	P-value
**Age group(years)**				
18-28	Ref		Ref	
29-31	1.07 (1.00, 1.14)	0.053	1.03 (0.96, 1.10)	0.422
32 and above	1.03 (0.95, 1.13)	0.437	1.00 (0.91, 1.10)	0.954
**Marital status**				
Single	Ref		Ref	
Married/cohabit	0.95 (0.90, 1.00)	0.063	0.96 (0.91, 1.01)	0.144
Divorced	1.07 (1.03, 1.11)	<0.001	1.03 (0.97, 1.10)	0.337
**Education level**				
No formal	Ref		Ref	
Primary	1.15 (0.92, 1.43)	0.230	1.18 (0.95, 1.46)	0.137
Secondary	1.13 (0.91, 1.41)	0.279	1.18 (0.95, 1.46)	0.144
Above Secondary	1.20 (0.94, 1.51)	0.138	1.28 (1.01, 1.62)	0.045
**Occupation**				
Employed	Ref		Ref	
Unemployed	1.00 (0.90, 1.12)	0.970	1.05 (0.93, 1.18)	0.410
Self-employed	1.05 (0.94, 1.16)	0.378	1.07 (0.95, 1.20)	0.262
**Number of dependents**				
None	Ref		Ref	
1-2	1.00 (0.88, 1.12)	0.961	1.01 (0.90, 1.13)	0.901
3-4	1.04 (.92, 1.18)	0.491	1.04 (0.93, 1.18)	0.488
5 and above	1.08 (0.96, 1.22)	0.204	1.07 (0.95, 1.22)	0.264
**Participation experience (number of trials)**				
1	Ref		Ref	
2 and above	1.12 (1.08, 1.15)	<0.001	1.10 (1.05, 1.14)	<0.001
**Income**				
0-$**59.82**	Ref			
Above $**59.82**	0.97 (0.90, 1.04)	0.346	0.96 (0.89, 1.03)	0.242

### Association between reasons/ethical perspective (23 items-full scale) and participant background characteristics

For the full scale with all items (financial and non-financial motivations), similar results were found. Married or cohabiting individuals had a significantly lower likelihood of a positive outcome compared to single individuals (APR (95% CI): 0.86 (0.79, 0.92)-P-value: < 0.001). Finally, participants who had taken part in two or more trials had a significantly higher likelihood of agreeing to the motivations for their research participation compared with those less experienced. (APR (95% CI): 1.12 (1.06, 1.19)-P-value: < 0.001) (Table 5).

**Table 5:** Association between reasons/ethical perspective (23 items-full scale) and participant background characteristics.

Characteristics	PR (95%CI)	P-value	APR (95%CI)	P-value
**Age group(years)**				
18-28	Ref		Ref	
29-31	1.10 (1.00, 1.2)	0.044	1.01 (0.92, 1.11)	0.843
32 and above	1.00 (0.88, 1.15)	0.953	0.95 (0.83, 1.08)	0.437
**Marital status**				
Single	Ref		Ref	
Married/cohabit	0.85 (0.79, 0.91)	<0.001	0.86 (0.79, 0.92)	<0.001
Divorced	1.07 (1.03, 1.12)	<0.001	1.05 (0.97, 1.15)	0.218
**Education level**				
No formal	Ref		Ref	
Primary	1.23 (0.92, 1.65)	0.164	1.28 (0.97, 1.69)	0.082
Secondary	1.20 (0.89, 1.6)	0.213	1.28 (0.97, 1.69)	0.082
Above Secondary	1.18 (0.84, 1.66)	0.344	1.29 (0.92, 1.81)	0.140
**Occupation**				
Employed	Ref		Ref	
Unemployed	0.97 (0.84, 1.12)	0.718	1.04 (0.89, 1.20)	0.636
Self employed	1.06 (0.93, 1.20)	0.417	1.05 (0.91, 1.21)	0.483
**Number of dependents**				
None	Ref		Ref	
1-2	0.91 (0.81, 1.04)	0.159	0.94 (0.83, 1.07)	0.343
3-4	0.98 (0.86, 1.12)	0.770	0.99 (0.88, 1.14)	0.998
5 and above	1.01 (0.87, 1.16)	0.943	1.01 (0.87, 1.17)	0.910
**Participation experience (number of trials)**				
1	Ref		Ref	
2 and above	1.17 (1.10, 1.24)	<0.001	1.12 (1.06, 1.19)	<0.001
**Income**				
0-$**59.82**	Ref			
Above $**59.82**	0.97 (0.89, 1.07)	0.557	0.96 (0.88, 1.05)	0.394

## Discussion

This is one of the first studies that focuses on the importance of both financial and non-financial motivations for research participation in prevention trials among Tanzanian healthy volunteers. This study reveals that being single and having an informal education is associated with financial incentives as motivators for research participation. Also, participants who took part in two or more trials have significantly higher non-financial participation motivations compared to those with less experience. While financial incentives are important, they may not account for other non-financial reasons such as a felt duty to participate, access to medical follow-up, quality information, disease control, and contribution to one's society and neighbor.

It was not surprising that more than a third of our participants indicated financial incentives as a motivator for their research participation. However, only those who were single were nearly two times more likely to report financial compensation as a motivation as did those with no formal education. The finding suggests that financial incentives are a stronger motivator for research participation among single individuals and those with no formal education. These groups may have fewer financial opportunities, making compensation more appealing. Our findings support the work by Fisher et al. (2018) who reported that more than a third of their participants felt that financial compensation kept them economically “afloat” and those with a high school degree or less were more likely to find benefit in compensation [17]. Thus, compensation may serve as an ethical necessity for one's day-to-day livelihood and to meet individual and family needs, however limited the amount. This also highlights the ethical importance of reducing financial burdens for participants, particularly in economically constrained settings [18,19].

Financial compensation is particularly relevant in Tanzania, where approximately 26 million people live in extreme poverty (<$1.90 per day) [20,21]. Guidelines that strictly regulate financial compensation in clinical trials may inadvertently limit participation among economically disadvantaged groups, potentially exacerbating health inequities. Future research needs to qualitatively understand the perceived value of financial compensation for those who are single, unemployed and with less education and its effect on related outcomes as well as to quantitatively assess combined factors (e.g., marital status and education) and their importance in decision-making. Moreover, it is difficult to know whether these groups would have participated without financial compensation as we did not specifically ask them whether receiving compensation was their main driver for participating in the prevention trial although some authors found this to be the case for their population of interest [3,22].

Interestingly, participants who had taken part in two or more trials were also more inclined to view financial compensation as a motivational reason to participate in prevention trials. This may be due to previous exposure to financial incentives and perceived benefits such as free healthcare, cash rewards, or in-kind compensation as also documented [22,23]. It is not clear whether repeat participation in clinical trials normalizes financial compensation as a reasonable expectation rather than an ethical, but future research should examine this concern.

The quality of the information provided by investigators as well as planned medical follow-up, and psychological benefits, such as a sense of personal fulfillment, were also key factors influencing our participants’ decisions. A large majority of participants also indicated a “duty” to participate and some also agreed that they felt a “duty” to the person who recruited them. This finding supports research by Reed et al. (2017) who studied vulnerable female sex workers in India and who reported on community pressures because madams or fellow female sex workers who took them to participate in the research project also required a commission or reimbursement [24]. This leaves unanswered questions about voluntariness and pressures that our participants might have experienced from the principal investigator, family, friends or the individual(s) who recruited them to the prevention trial. We concur with Reed et al. (2017) who stated that “community support of research projects may also create pressure to participate.” (p. 4).

Transparency, adequate communication, and alignment with participant expectations are essential in the ethical conduct of research [1]. Future studies should explore how compensation interacts with altruism and other perceived personal benefits to refine ethical compensation models in the developing world. In this study, altruistic motivations—such as disease control and community contribution—were more strongly agreed to than financial motivations. However, financial compensation remained an important motivator among participants with higher incomes within the country (although less than $60 and not statistically significant), greater numbers of dependents, and those who were self-employed. These insights can potentially inform future recruitment and retention strategies while ensuring ethical guidance on compensation for research participation.

This study has several limitations. First, participants were recruited from specific prevention trials, and those who declined participation (n = 104) may have had different perspectives and reasons for their initial participation. Thus, there is the potential for selection bias. Future research should assess differences between diverse participants and those who declined. Second, the study assessed research motivations at a single point; however, perspectives on the role of financial compensation may change over time depending on the procedural aspects of the trial and other personal challenges of those who volunteer. A longitudinal approach would provide deeper insights. Third, findings may not generalize to other research settings or higher-risk studies. Fourth, all study participants were female and while it is important to understand the motivations of women to participate in research, male perspectives would also provide additional valuable insights. Finally, we were limited by only focusing on socio-demographic variables. Future research should examine clinical and study team variables that might be important in volunteers’ decision to participate in prevention trials, especially since some of our participants indicated a felt need to participate with an obligation to the person requesting their participation. Additionally, while both studies in this research were treated as prevention trials, the type of study (e.g., HIV vs. calcium supplementation) may also impact participant motivations and warrants further investigation. However, the study's findings contribute to a broader ethical discussion on the role of financial and non-financial compensation in prevention trials from healthy volunteers’ advantage point of view. Given the study's quantitative approach, future research should include qualitative methods to explore participant motivations more deeply. Also, we did not consider the potential influence of the COVID-19 pandemic, and it remains unclear whether the pandemic affected participants’ decisions regarding financial compensation, altruism or access to health care benefits. Future research should examine how such external factors may shape participant motivations.

## Conclusions

Financial compensation is important to certain subgroups of participants. Further research is needed to better understand the importance of compensation from these groups. Although financial compensation was widely viewed as beneficial, its role varied among participants, with many emphasizing altruism and trust in research oversight over monetary incentives. These findings underscore the need for balanced and transparent compensation frameworks that address participants’ financial realities while safeguarding ethical integrity and discussing other benefits of study participation beyond compensation.

## Supporting information

S1 File Inclusivity in global research questionnaire 3. (PDF)
